# Blockchain for Electronic Vaccine Certificates: More Cons Than Pros?

**DOI:** 10.3389/fdata.2022.833196

**Published:** 2022-07-08

**Authors:** Raphaëlle Toubiana, Millie Macdonald, Sivananda Rajananda, Tale Lokvenec, Thomas C. Kingsley, Santiago Romero-Brufau

**Affiliations:** ^1^Department of Biostatistics, Harvard T.H. Chan School of Public Health, Harvard University, Boston, MA, United States; ^2^University of Queenland, Saint Lucia, QLD, Australia; ^3^Institute for Applied Computational Science, Graduate School of Arts and Sciences, Harvard University, Cambridge, MA, United States; ^4^Department of Medicine, Mayo Clinic, Rochester, MN, United States; ^5^Department of Biomedical Informatics, Mayo Clinic, Rochester, MN, United States; ^6^Department of Otolaryngology - Head and Neck Surgery, Mayo Clinic, Rochester, MN, United States

**Keywords:** blockchain (BC), electronic vaccination record, electronic vaccine certificate, cryptography, COVID-19, clinical informatics

## Abstract

Electronic vaccine certificates (EVC) for COVID-19 vaccination are likely to become widespread. Blockchain (BC) is an electronic immutable distributed ledger and is one of the more common proposed EVC platform options. However, the principles of blockchain are not widely understood by public health and medical professionals. We attempt to describe, in an accessible style, how BC works and the potential benefits and drawbacks in its use for EVCs. Our assessment is BC technology is not well suited to be used for EVCs. Overall, blockchain technology is based on two key principles: the use of cryptography, and a distributed immutable ledger in the format of blockchains. While the use of cryptography can provide ease of sharing vaccination records while maintaining privacy, EVCs require some amount of contribution from a centralized authority to confirm vaccine status; this is partly because these authorities are responsible for the distribution and often the administration of the vaccine. Having the data distributed makes the role of a centralized authority less effective. We concluded there are alternative ways to use cryptography outside of a BC that allow a centralized authority to better participate, which seems necessary for an EVC platform to be of practical use.

## Introduction

### The Rise of COVID-19 Electronic Vaccine Certificates

The requirement of proof-of-vaccination to COVID-19 is gaining traction in government agencies and the private sector, despite vocal opposition. The European Commission on December 21st, 2021 created regulations around the use of European Union Digital COVID Certificates (EUDCC) (EU Digital COVID Certificate, 2022). These regulations apply to all nations (non-EU included) that choose to adopt the EUDCC. Its primary use is to open travel between EU countries, but some nations are using it domestically to control entry to public places such as restaurants or sporting events. As of February 1st 2022, 42 countries are already connected to the EUDCC, and many more are considering joining (EU Digital COVID Certificate, 2022). The EUDCC uses a technology called distributed identity. The United States (US) federal government has taken a more limited role in regulating and mandating proof-of-vaccination through EVC platforms. This has left the responsibility to the private sector and state governments. Employers such as airlines, hospitals, and restaurants are increasingly requiring proof-of-vaccination for their patrons and employees (Eldred, 2021). Other non-EU countries are also evaluating EVC technology platforms to use domestically.

### Blockchain Technology as a Solution

Blockchain has been a commonly proposed technology solution for COVID EVC platforms (Mithani et al., 2021). Although awareness of blockchain has increased because of the rise of digital currency such as Bitcoin and Ethereum, the majority of the public and decision makers have little understanding of the technology, especially in non-currency-based uses. Moreover, despite vocal opposition to proof-of-vaccination measures, it seems likely some versions of them will stay and become more widespread as COVID becomes more endemic, especially if COVID remains a deadly disease in those unvaccinated.

Blockchain use in EVCs is commonly proposed but there is a paucity of literature or real-world examples of its use for this purpose. As pressure increases for decision makers to choose amongst the various technology options, the authors of this paper thought it was important to review this topic.

### Ten Important Characteristics of an EVC Technology Platform

As governments and the private sector are evaluating EVC platforms for deployment there are multiple considerations. Through discussion, our team identified 10 key considerations: **(1) data privacy and security** (patient health information, demographic data, location, etc), **(2) data verifiability and fidelity** (data remains auditable and accurate over time), **(3) data retrievability** (data can be queried and retrieved with accuracy and within a timeframe that is useful for its application), **(4) technology accessibility** (how easy it is for the public to access it as users), **(5) equitable (**regardless of socioeconomic, racial, or cultural differences), **(6) interoperability** with other public health and healthcare system information technology, **(7) scalability** (to be broadly available to the public within a short time period) **(8) cost effective** to maintain and operate **(9) potential for public adoption** (important factors include understandability, trust, and public perception of the technology), and **(10) feasibility** of development and operationalization (e.g., prior examples of the technology platform being successfully deployed in similar contexts).

## Background

### Databases

A data storage application like an EVC system would traditionally use a database (generally what is called a relational database) to store patient and vaccination data. A relational database can be compared to a Microsoft Excel or Google Sheets document - data is stored in tables with rows of entries similar to a spreadsheet, and may contain multiple, possibly interlinked tables similar to the tabs in an Excel or Sheets document. Data can be retrieved from the database by writing queries in the appropriate query language, similar to the functions that can be used with Excel and Sheets. There are other types of databases that do not use blockchain technology, and the main benefit of databases is that they can also be optimized for specific use-cases, such as minimizing the size of the data and increasing the speed of updating or querying the database.

Theoretically, any kind of data can be represented in a database in any way, with any kind of relationships between different pieces of data. For example, for an EVC, there might be one table where each row contains the full private data of a patient and a vaccination they received. Alternatively, for a vaccine that requires multiple shots, data that is duplicated between each entry, such as a patient's details, could be entered into its own table which can then be linked to a second table that contains only the data for each shot. This way, the amount of data stored for each patient is reduced, and therefore so is the overall size of the database. This can lead to various improvements to the overall system, including the hard drive space required to store the database.

Generally, the security of the data in a database depends on the security of the systems it is connected to, unless the data itself is encrypted (see glossary). For example, most applications that use a database would have a user interface (UI) to make it easier for users to view and update the data in the database. Permission systems (such as usernames and passwords) can be used to control who can do what with the database data - for example, perhaps anyone with a login can read the data entries that pertain to themselves, but only some people can add or change data. The security of such an application then depends on factors like who has permission to do what operations, and how easy it is for a malicious entity to gain access to the database (e.g., by hacking the system or stealing login information from a user and using it to access the data *via* the user interface). Cryptographic techniques are commonly used at various points in an application in order to add layers of security.

## What is Blockchain Technology

Blockchain is a distributed ledger technology for storing and transmitting information. Its main characteristics are transparency, security, and decentralization (operating without a centralized control body) of both data and authority (Cawrey, 2021). A common application is money transfers that can be performed without the need for trusted third parties or banks. This is how Bitcoin or Ethereum work: thanks to blockchain, there is peer-to-peer (P2P) review that permits direct transfers between individuals.

The blockchain can therefore be compared to a public, anonymous and unforgeable accounting ledger. We can also think of this technology as a way to securely store private information such as vaccination records. In this section we describe what's known as a public blockchain, which is the original design by Nakamoto (2008). There are other variations of blockchain called “permissioned blockchains” that we will describe in the next section.

The first step is to initiate the transfer.

Let's say Mike wants to do a transaction with Santiago. If we consider Bitcoin for example, Mike would like to transfer money to Santiago; in that case we would have a record that says: “Mike pays Santiago 2 Bitcoins (transaction signed by Mike).” If we consider vaccination records, we could record the vaccination similarly: “Mike vaccinates Santiago (transaction signed by Mike),” with Mike being a vaccinator. A vaccinator is anyone approved to administer the vaccine, often a licensed healthcare provider or a public health official.

In step two, the transaction is sent to the network, composed of all the people using the blockchain, for verification. The first verification concerns the identity of the individuals involved in the transaction: is it really Mike that wants to do the transaction with Santiago?

**Figure 1:**
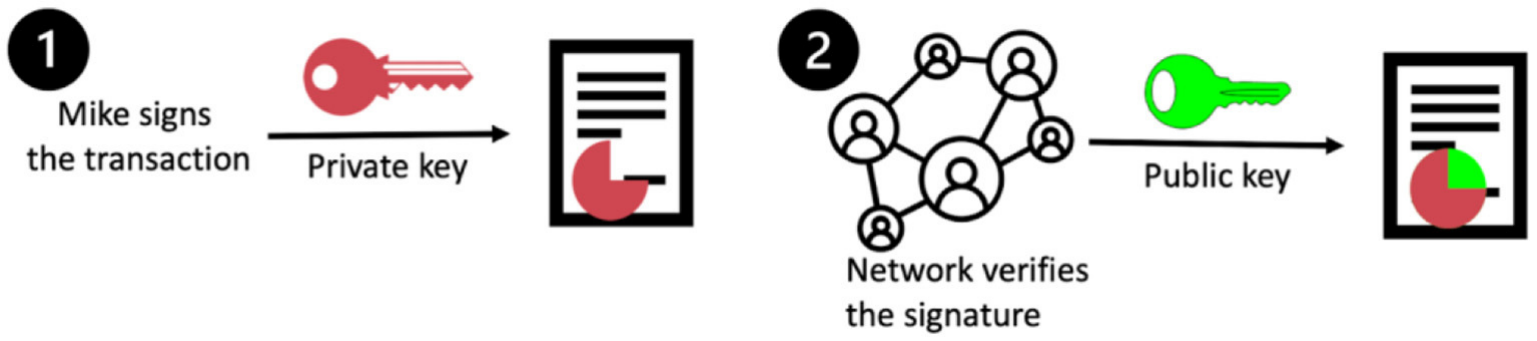
Verification of Mike's identity.

How does this validation step work? Mike has to sign the transaction with an electronic signature called a private key. Only Mike has access to this key. The rest of the network has a public key that can only be used to decode Mike's private key. When the transaction is sent by Mike, several people in the network will verify that their public key decodes Mike's private key (Figure 1). If the public key doesn't decode Mike's private key, it means that it is not really Mike that sent the transaction. The transaction is thus canceled.

In the case of money transfers, the verification consists of verifying the identity of Mike with his electronic signature, as explained above, and verifying if Mike has enough money on his account to send to Santiago. In the case of vaccination records, one could envision a similar verification process using two keys to verify the identity of the parties.

The transaction is approved only if more than half of the people on the network accept it. This way, since there is a vast number of users, it is very unlikely that a compromised transaction will be approved.

**Figure 2:**
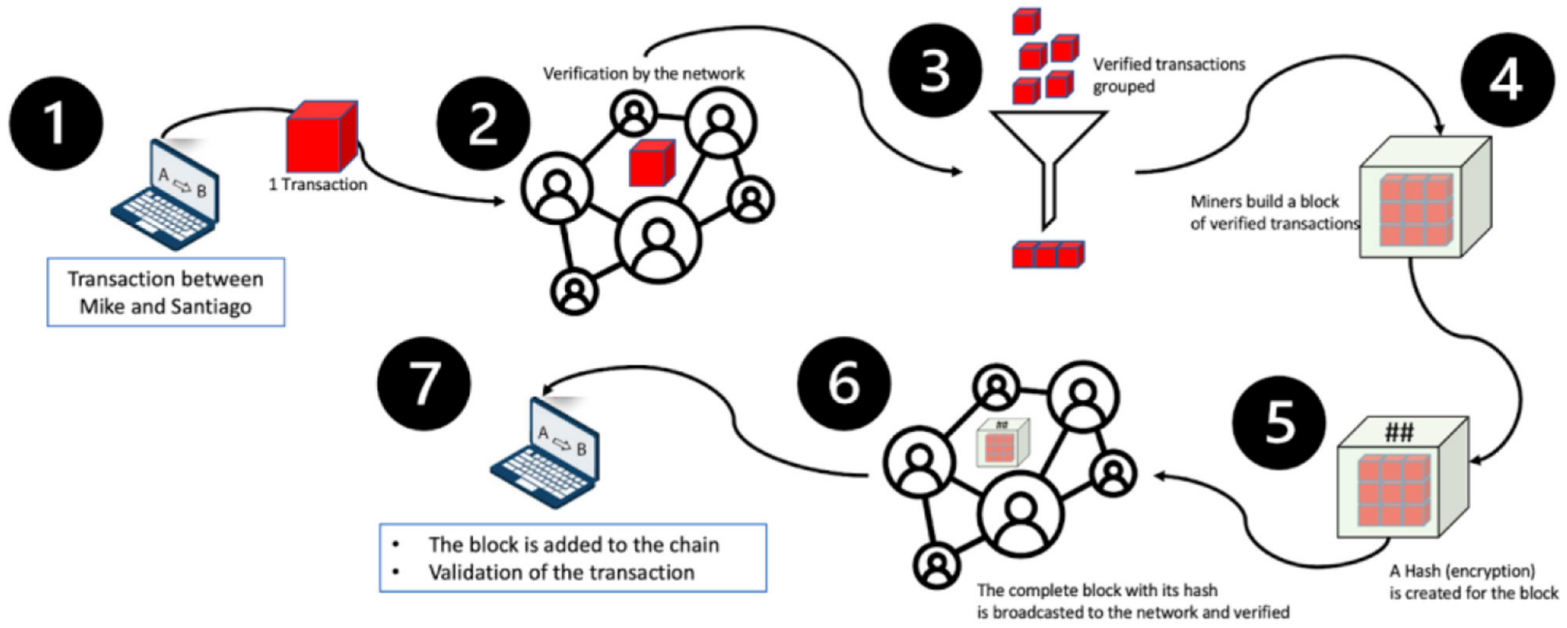
Process of adding a transaction to the blockchain in 7 steps. The ^*##*^ indicates the hash that was created for the block between steps 4 and 5.

Once the transaction is verified by the network, it is grouped together with other transactions to form a block (Figure 2, step 3).

On step four (Figure 2), a block is built for the group of transactions.

In Bitcoin and other proof-of-work systems, the “validators” of the chain, also called “miners,” must spend computational work to find the solution to a mathematical problem, and that solution links the block to the chain. In systems using proof-of-stake or proof-of-authority, the miners only need to produce a digital signature that authenticates it to the network.

Once the block is validated, a timestamp is added to the block, i.e., the approximate date and time when the block was found.

**Figure 3:**
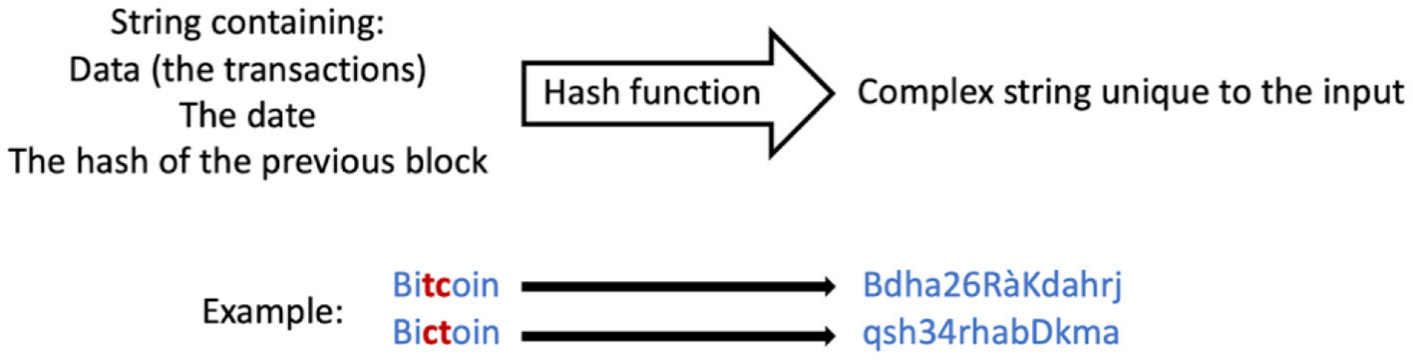
Hashing.

Step five (Figure 2) is called hashing. Each block has an identifier, which is a unique cryptographic fingerprint, resulting from the hash of the data that this block contains: the transactions, the timestamp and the hash of the previous block. If someone attempts to modify the information stored in a block, the hash will change drastically, and the fraud will be detected (see Figure 3).

**Figure 4:**
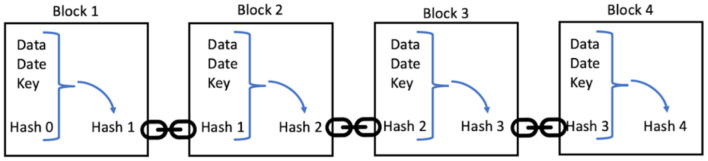
Schematic of a Blockchain.

The block is then broadcast to the network and is verified one last time before being added to the chain. We call this technology blockchain, because each block of transactions is linked to the previous one through the hash, as shown in Figure 4.

## Permissioned Blockchains

In the previous section we have described the general functioning of blockchain technology. However, there are multiple variations, which can change critical aspects of the technology.

In general, there are three types of blockchains: public, consortium, and private (Zhang and Lin, 2018). Public blockchains such as Bitcoin allow anyone to participate: there are no restrictions on who can read or write to the blockchain. Consortium blockchains are permissioned blockchains where a consortium of entities are able to validate blocks. Access to the blockchain may vary between public or restricted (e.g., *via* APIs). Private blockchains are permissioned blockchains where a single entity has complete authority over the network and that entity fully controls both read and write permissions.

In the context of vaccination records, public blockchains will likely not suffice since vaccination records in the chain must be trustworthy (i.e., they should be added to the chain by a trusted medical entity). This then naturally leads to a private or consortium blockchain, where the ability to add to the chain and validate blocks can be restricted to only trusted entities, such as vaccinators (doctors and professionals in the medical community). In this scenario, we can imagine a certain trusted entity, such as the Health Ministry of one or several countries, having control over who is allowed to add vaccination records to the blockchain. A system like the European Union Digital Covid Certificate allows any of several countries to add vaccination records.

### Proof-of-Work Validation

We have described how and when a block is validated. After this occurs, it is then added to the blockchain (Step 4 on Figure 2). However, there are many different consensus algorithms for validating blocks. The most popular, due to its use in Bitcoin and the way it incentivizes participation, is the proof-of-work algorithm.

In proof-of-work blockchains, a block is validated by performing a task that is computationally expensive, but easy to confirm. For example, in Bitcoin this task is finding a sequence when added to the block that will result in a hash that ends in a certain number of zeroes. This requires miners to use trial-and-error to find a sequence that will result in a certain hash. However, once that sequence is found, it is very easy to confirm that its hash has the required number of zeroes. Proof-of-work systems often need to provide an incentive to the agent who solved the problem. In currency-focused blockchains, this is easily solved by rewarding that agent with a certain amount of currency.

However, in an EVC system there isn't a clear reward that could be provided to the agent that validated the block. For these reasons, a proof-of-work validation algorithm would not be appropriate for this application, and other validation systems would need to be used. An algorithm which relies on a majority consensus between parties may be best, and especially, if used in a permissioned blockchain system, where the various entities are trusted.

In Table 1 we summarize the differences between a public blockchain and a permissioned blockchain. An EVC system would likely use a permissioned blockchain.

**Table 1:** Differences between public and permissioned blockchains.

**Property**	**Public**	**Permissioned**
Access restrictions	No restrictions inherent to the blockchain	Ability to read and write data to the blockchain is controlled
Trust	Doesn't require trust between agents in the network	Requires trust, due to agents having different read, write and validation permissions
Risk of takeover by majority of authoritative nodes	Anyone can join the network and validate transactions	Only some nodes are authoritative (can validate transactions)
Security	Malicious entities can easily gain access, and data is public	Permissions control who can do what, including viewing the data
Validation	Anyone can validate blocks, but validation is computationally expensive, so an incentive is generally needed	Trusted entities can be assigned the duty of validating blocks which removes the need for an incentive
Consensus algorithm	Can operate in an environment with low trust between entities, and may need to handle faults and malicious entities	Trust allows the consensus algorithm to be simplified

In some ways a permissioned blockchain is more similar to a traditional database compared to a public blockchain. For example, fewer authoritative entities means that an entity or group of entities could theoretically gain authority more easily, allowing them to block new transactions and rewrite their past transactions. However, in a permissioned blockchain, like in a traditional database, those entities need to be externally permissioned, which increases security.

However, a blockchain-based application will generally have more components than just the blockchain, such as user management and other data storage. A permissioned blockchain may allow for security trade-offs to be made elsewhere, such as choosing a less secure but faster consensus algorithm.

### Considerations for Cooperative Applications

Decentralized authority may be an appealing solution when multiple entities are collectively using a system and each one is unwilling to let others have more authority over the system (such as countries sharing a common vaccination record system). This then could incentivize additional entities to join the blockchain.

However, a major hurdle for using blockchain technology on such a large scale is agreeing on a common protocol for the chain. These include the consensus mechanism, privacy standards, incentives for maintaining the chain, and managing write access to the chain. In addition, there has to be some level of trust that the other entities are managing their write access to the chain properly and those records can be trusted.

Some technical designs using consortium blockchains for EVC have been described (Haque et al., 2021).

In the case where multiple countries share the same blockchain, a consortium blockchain could theoretically be employed. This would allow each country to control the permissions to their respective medical institutions to write to the chain. Since no one country would have complete authority over the blockchain, the core benefit of decentralized authority would be preserved.

With regards to suitable blockchain platforms, Bitcoin and Ethereum are public, not consortium. Other platforms such as Multichain, Hyperledger Fabric and Hyperledger Sawtooth are likely more appropriate (Chowdhury et al., 2018, 2019).

## Differences Between Data Storage in Blockchain and Databases

The biggest difference between blockchain and other types of distributed ledger technologies is the use of cryptographic techniques to add a layer of security to the data. While cryptography is often used for secrecy, in the context of blockchain the technology is used to make it significantly harder to change the transaction history, as described above. This is how cryptocurrency got its name. It is currency that is traded on the blockchain, many of the advantages of which come from the cryptographic techniques it utilizes.

As mentioned above, databases are based around storing data in tables with various methods for optimizing a database. This flexibility, especially combined with the various innovations in database technology and other fields over the last few decades, means there is very little to which databases are not suitable, with the right configuration.

Blockchain technology, in comparison, is designed to store individual data entries in a chronological manner. Innovations such as Ethereum have greatly improved what kinds of data can be stored on a blockchain, but the chronological nature of the technology and the fact that each data entry is independent of any other entry are core to blockchain.

With cryptocurrencies such as Bitcoin, people who use the currency do not directly access the blockchain to make transactions. Each user has a “wallet” which contains a list of their private keys, usually combined with a software interface with which users can manage keys and make transactions (Frankenfield, 2022). The data within a wallet is not stored on a blockchain. Instead, there are various data storage methods that are used, and one common option is a traditional database.

## Analysis of Blockchain Technology for EVC Use

### Pros and Cons of BC Compared to Traditional Databases

Many blockchain platforms now exist, but most are designed for specific use cases or are too early in development or adoption for a use case as important as EVCs. The following therefore generalizes blockchain systems, based mainly on popular platforms Bitcoin and Ethereum. On Table 2 we provide a summary of the characteristics of blockchain and how they relate to the EVC use case.

**Table 2:** Properties of blockchain and how they relate to the EVC use case.

**Property**	**Advantage**	**Disadvantage**	**Mitigation**	**Counterfactual**
Decentralized authority (public blockchain)	**Safe operations** of applications **Incentivize co-operation** of shared authority	**Agreement** on protocols, etc. Can't control who has access	Use **private or consortium permissioned** blockchain	Standard databases can be **permissioned**
Decentralized data storage	**Less risk of data loss** with redundancy of data	The dataset for each authority can become **extremely large** blockchain	**Limit which entities** require the full blockchain **Limit on-chain** data storage	Minimization of data loss risk in traditional databases through **backups or other redundancy methods**
Immutability, data handling	**Improved data security** thanks to limited data operations (create, read)	**No updates or deletion** of data **Overhead** introduced to create and read operations	Data **can not be erroneous**, or **policies** must be created for changing chain history	All operations **allowed** in databases and can be **controlled** through permissions. Possible **performance optimization**
Timeline verification	**Reliable verification** of timeline	N/A	N/A	**Similar timeline verification** functionality with database encryption methods
Resource usage (energy and computation)	Usage **controlled by blockchain implementation** choices, e.g., consensus algorithm	**Significant energy consumption**, particularly of popular blockchain properties	**Architect** blockchain to reduce resource usage, e.g., choice of less energy-intensive **consensus** algorithm	Databases can be **optimized to minimize resource usage**
Pseudonymous identities	**Tracking** of transactions by entities	IDs (usernames) **can not be linked** to real-world identities without integration with **external systems**	Integrate with **external identity systems**	Standard databases can use **any identity verification system** and completely control the creation of identities
Performance	**Validity** of data and **ordering** thereof ensured	**Block validation speed** affects performance	**Carefully select properties** such as block size limit	Standard databases are **faster and more optimized**

*Bold is for emphasis*.

### Decentralized Data Storage

Decentralized data storage means that, theoretically, every node would have a complete copy of the blockchain. However, blockchain data can grow quickly to gigabytes or even terabytes of data. For example, as of January 20th 2022, the blockchain size of Bitcoin was 386 GB for its 704 million transactions (Blockchain Charts). The full Ethereum chain was 1178.68 GB (Ethereum Chain Full Sync Data Size, 2022).

The full blockchain is required for authorities who validate blocks, but usually not required just to create transactions. It is also unrealistic that every entity would be willing to store the full chain. Therefore, these blockchains can create light nodes, which only store the data necessary to create transactions and rely on full nodes for other data as well as validation (Wackerow, 2022).

The blockchain size scales with the number of transactions and the data size of each transaction. Databases scale in a similar way, but as a more mature technology are optimized to reduce the impact. Data redundancy is another benefit of decentralized data but can also be achieved with databases using backups.

For context for the EVC use case, the population of the USA is 329.5 million with 551 million doses given. The population of the European Union is 447 million with roughly 848 million doses given (Daily COVID-19 vaccine doses administered, 2021; Ritchie et al., 2022). These vaccinations have been done in the last year, compared to Bitcoin's transaction history which goes back to 2009. This means that not only would vaccination records quickly exceed the size of Bitcoin transaction history, it would also present problems with record entry speeds.

Blockchain systems tend to limit how fast entries can be added by controlling how long or how big blocks can get. For example, Bitcoin is designed so a new block is mined every 6–10 min. This restriction on the system may be a significant problem with EVCs, whether they are set up at the beginning of vaccinations or, like now, potentially having to catch up with a significant number of past vaccinations.

### Immutability, Data Handling and Performance

Databases support operations to create, read, update and delete (CRUD), and who performs each of these operations can be managed with permissions. Blockchain only supports create and read operations. As past transactions cannot be easily changed, this theoretically creates an immutable record. Rewriting the chain is technically possible, but extremely difficult. It would require changing past transactions, propagating the changes through the chain, then getting majority acceptance from the authoritative nodes. This would require recomputing blocks, which may be costly and slow. The majority agreement may also be difficult. Other options for changing the chain may be viable but depend on the specific blockchain implementation.

Databases can be optimized for the most used operations. Blockchain's “create” and “read” operations are slower due to the overhead of the validation and consensus mechanisms. Bottlenecks can also happen, such as block validation delays slowing transaction processing.

Databases are also designed to allow for any data to be queried based on any relationship between the data points. For example, an EVC database could likely be easily queried for “one patient's records,” or “everyone vaccinated with a specific vaccine lot.” Blockchain data is not designed to be queried in this way, as it is structured based on individual transactions and metadata about the entities doing transactions. It is possible to replicate such queries with blockchain technology, but due to it not being designed for such purposes this requires additional effort to implement and compute.

Whether immutability is beneficial for an application can depend on the risk of human error. For instance, is the data generated by a trusted program, or is it entered by humans who may make mistakes? If reading data very soon after it is created is important, databases may be preferable to blockchain.

Some existing blockchain applications try to get around some of the limitations of blockchain by using a combination of blockchain and databases. This requires careful implementation. A recent incident with OpenSea, a blockchain application that allows users to trade in images and other media, which used a hybrid blockchain and database approach to avoid Ethereum's high transaction fees. A bug was found where the blockchain and database got out of sync. This allowed an attacker to buy several items at an older, lower price, then sell them at the more recent price for a substantial profit (Cimpanu).

### Timeline Verification

A major advantage of blockchain is that transaction validity and order can be easily verified. This is due to it being an immutable and chronological ledger. Databases can store timestamps for entries, security techniques can be applied to achieve immutability, and there are methods of encrypting database information to provide similar functionality.

In the case of EVCs, the specific order of the records is not critical. For example, it does not really matter whether Sue was vaccinated before or after Mary.

### Pseudonymous Identities

An EVC system will require integration with real-world identification systems. A common example is using Social Security Numbers in the US to link the blockchain records with real-world people. This would apply to vaccinators, patients, and anyone else involved. There must also be checks to ensure individuals are not duplicated in the system.

Implementing these required checks in the blockchain system may be difficult for the same reasons querying data is difficult. Additionally, the existing identity systems are traditional databases, and integration with a blockchain-based system would add complexity and challenges.

### Resource Usage

Blockchains can require a significant amount of computation and energy. Different blockchain implementations require different amounts due to factors like choice of consensus algorithm. In proof-of-work verification, nodes race to complete the computation of each block for a reward, but as a winner-takes-all contest, energy used by the losing nodes is wasted. Other consensus algorithms tend to use less energy (Chowdhury et al., 2018), thereby lowering the energy cost of the entire system.

Another consideration is the resource usage of everyone using the blockchain application. Because of its distributed nature, all full nodes who are capable of validating transactions. This requires each entity to have a computer storing the full blockchain and capable of validating nodes, which most likely must run continuously. This requirement may affect adoption in the case of EVCs, as it is an added cost and burden on those entities who would have authority to validate blocks. Light nodes at least must only store part of the blockchain, and do not need the computation ability to validate nodes. So careful organization of who requires a full node and who can use a light node can minimize this distributed cost.

Databases, in comparison, due to their centralized nature, only use the energy required to run their servers (including those used for backups) and external systems such as air conditioning (Sedlmeir et al., 2020). Users of the application would connect to it *via* the Internet, so no special machines or systems are needed. This also allows for low-cost backups that can be performed routinely but do not require to be constantly connected and computing.

### Hype and Public Opinion

Blockchain, with regards to its use in cryptocurrencies, NFTs, and games, has been appearing in the news more often in recent months and years. It is a technology that is drawing a lot of attention and is often described as being “hyped” (Litan, 2021), meaning that the amount of attention and public expectations may surpass its actual delivery of progress. There have been reports of publicly-traded companies adding the term “blockchain” to their name and having their shares surge (What is in a name UK stock surgers 394% on blockchain rebrand, 2017). This points toward significant expectations associated with the term, regardless of its actual feasibility.

However, as with any novel term, its valence in the public opinion can quickly turn. For example, several companies in the software and gaming industries announced blockchain-related projects near the end of 2021, usually receiving mixed feedback from the general public. For example, when the CEO of Discord, a popular chat program, hinted at blockchain integration, there were supporters but also many users who were publicly against the move on Twitter, Reddit and Discord's own forum, and an unknown number canceled their paid subscriptions in protest (Orland, 2021). Molly White's timeline of problems with “web3” (a catch-all term for blockchain-based innovations), while focused on negative news, is a good indicator of what is happening in the space, especially in terms of its effects on the general public (White, 2022). It highlights that scams and hack are abundant in the web3 sphere, and many people are suffering losses, usually monetary, because of blockchain-based applications.

A question then, regarding adopting blockchain for EVCs, is “Will the public trust their data is safe on a blockchain-based solution?” Blockchain is known for being difficult to understand, not helped by the complexities around all the variations and different use cases it can be used for. If public opinion of the technology - informed or otherwise - becomes negative, will people be willing to have their private medical data stored using such a technology?

### Assessment of Blockchain for EVC

In Table 3 we summarize our assessment of the comparison between blockchain technology and traditional database solutions regarding the 10 key considerations presented in the introduction. As can be seen, blockchain only seems superior in the Data verifiability and fidelity domain, with all other aspects being either clearly inferior, equivalent, or uncertain.

**Table 3:** Comparison of blockchain and alternative technologies regarding EVC requirements.

**EVC platform technology feature**	**Optimal blockchain configuration compared to alternative technology solutions**	**Comments**
Data privacy and security	**Equivalent or uncertain** based on current information	Both blockchain and standard databases can use similar cryptographic techniques [Transparent data encryption (TDE), 2022].
Data verifiability and fidelity	**Superior**	Harder to forge records without leaving a trace of it in blockchains
Data retrievability	**Inferior**	Blockchain's data structure is not designed for flexible data queries, databases are
Technology accessibility	**Equivalent or uncertain** based on current information	Depends on the front-end design and not much affected by the underlying data storage technology
Equitable	**Equivalent or uncertain** based on current information	Same as above. Mainly depends on accessibility.
Interoperability	**Inferior**	Blockchain is a less mature technology, and by design harder to modify? combining data registries or changing data standards is much harder
Scalability	**Inferior**	Traditional databases can be more easily scaled in transaction rate and storage
Cost effectiveness	**Inferior**	Blockchain's distributed nature makes it more costly to maintain. Traditional databases have been optimized for efficiency.
Potential for public adoption	**Equivalent or uncertain** based on current information	As a novel technology, public perception of blockchain can change quickly
Feasibility	**Inferior**	Blockchain is a less mature technology compared to time-tested database solutions.

*Bold is for emphasis*.

## Current Blockchain-Based EVC Solutions

Some existing EVC solutions do claim to be using blockchain as part of their technology. A recent review by Mithani et al. (2021) listed eight such applications, including IBM's Digital Health Pass. However, most of these solutions have not made public the technical details of how blockchain is used. In fact, the solutions proposed in this article published in March 2021 are not operational today. Some of the webpages are not even functional. Raising the question whether would the projects are still active?

For these solutions, the question remains of whether blockchain is really a key part of the technology, or if the name is being used for the “hype factor.” Given the lack of transparency it is hard to estimate the number of truly functional blockchain platforms in use for EVC, but from our teams estimate it appears to be none.

## Discussion

In this paper we have described the conceptual framework of blockchain technology as it could apply to storing electronic vaccine certificates (EVC). We have also discussed some of the advantages and drawbacks. Overall, blockchain technology seems to have more cons than pros for this use case. In line with our assessment, some widely-respected cyber-security companies have also assessed that blockchain is not necessary for EVCs, taking the example of the European COVID certificates system (Schubert, 2021).

A recent review of blockchain applications for COVID-19 (Ng et al., 2021) found that “vaccine passport monitoring” was one of the most common applications described in blockchain papers. However, most papers were limited to the technical description or reports of technical performance. Several blockchain system designs for vaccine supply management have also been described (Peng et al., 2020; Yong et al., 2020; Antal et al., 2021).

There have been other attempts to use blockchain technology for the storage and access to vaccination records using what is known as “smart contracts” (Zhao and Ma, 2022). In these approaches, the common idea is that the vaccination data (including vaccine certificates) is stored publicly but in encrypted form. The blockchain “smart contract” is then used to manage access to the key that would allow to decrypt the public data or a portion of it (Abubakar et al., 2021). This has been shown to significantly increase speed and convenience of data retrievability compared to scanning the blocks in the blockchain to find the vaccination information (Abuhashim et al., 2021).

As mentioned earlier, some of the main principles that inspired the creation of blockchain technology run counter to the EVC use case. For example, one of the key principles is decentralized authority. However, with vaccination records it makes sense to have one, or a few, central authorities who certify that an approved vaccine was administered. In a blockchain that stores information about money, the agreement in the network that a certain person has X amount can be enough to make that judgment meaningful. However, vaccines must correlate with an external event in the real world (the person's immunity status against a virus). That requires a central authority to determine, at least, that what was administered was a vaccine. This centralized assessment could be delegated to each “physician” agent in the network.

The aspect of blockchain technology that makes the most sense for the vaccination record use case, is the use of cryptography, which is closely linked to privacy. However, as we have discussed, a centralized or federated system to record and store vaccinations using cryptography can be designed without the use of blockchain, possibly using another distributed ledger technology. For example, a very simple system could store hashed records and make them publicly accessible. In the simplest form, there would be one hash per vaccination record. In this case the patient would go get their vaccine at a point of care and would have privileged access to the public record. After confirming the patient's identity, they would put information about the patient (e.g., patient full name and date of birth), the vaccine administered (e.g., vaccine name, provider, and lot number), and the date of administration, and create a hash with that information. Because this cryptographic hash is a one-way function that can't be tracked back, the hash can be posted publicly without loss of patient privacy. The provider would then upload this information into a public repository maintained by the authorized central agency (either the CDC or a similar organization). Then, to verify the patient's vaccination status, the patient would only need to present the information that was used to create the hash (which includes their identification), and the verifier could run it by the hashing function and compare to the public list of hashes posted in the trusted public repository. This hashing and comparison step could be easily automated into a phone app that would either read the patient's information from a printed vaccination card, or from a QR code that the patient would carry. There is a similar idea to that described in recent papers (Haque et al., 2021).

There are other questions that would need to be resolved almost independently of the technology used to store the vaccination records. There are several COVID vaccines available, with varying degrees of effectiveness. Ideally, the technology would store the information that is the most primary. In the case of an EVC, that's probably the record of which vaccine was administered, and when. This way, the rules of what constitutes a “fully vaccinated” patient can be flexible for different uses and can even be adjusted as more information becomes available. For example, if evidence becomes clear that vaccine efficacy wanes significantly with time, some countries may choose to include the time from the last dose in the definition of “fully vaccinated.” However, even in this scenario, a central body still needs to decide whether some vaccines are not considered effective enough to even include in the record.

## Conclusion

While blockchain has some useful applications, it does not seem to have clear advantages for electronic vaccine certificates (EVC) compared to more traditional database technologies. There is significant hype associated with blockchain that could be motivating its utilization for use cases in which it is not necessary. The existing EVC solutions that claim to use blockchain do not provide enough detail to assess whether blockchain is a core component of the system.

## Author Contributions

SR-B, RT, SR, and TL conceptualized the paper. SR, RT, TL, TK, and SR-B drafted the initial manuscript. MM significantly revised the manuscript critically. SR, RT, TL, and MM performed the review. RT composed the figures. SR-B and TK provided general guidance. All authors contributed to the article and approved the submitted version.

## Conflict of Interest

TK has a role in Pathcheck Foundation, a non-profit involved in the development of public health related technology that partially uses cryptography. The remaining authors declare that the research was conducted in the absence of any commercial or financial relationships that could be construed as a potential conflict of interest.

## Publisher's Note

All claims expressed in this article are solely those of the authors and do not necessarily represent those of their affiliated organizations, or those of the publisher, the editors and the reviewers. Any product that may be evaluated in this article, or claim that may be made by its manufacturer, is not guaranteed or endorsed by the publisher.

## Glossary

**Cryptography** is the study of techniques with which communications can be secured such that only the sender and intended recipient can understand the message. Encryption is a technique that is part of cryptography, where data is scrambled so that it is unintelligible, then sent to the recipient who knows how to unscramble it.

**Encryption** is the process of codifying the data so that it cannot be immediately read without an “decryption key”. The data is scrambled (as with a hash) and can only be unscrambled into an understandable form by using the decryption key. Data that is encrypted is more secure because, even if a malicious agent manages to access the data storage, they won't be able to read the data itself unless they also have access to the decryption key.

**Hashing** is a method of scrambling data that is often used in encryption as it creates a fixed-length series of characters which are usually shorter than the original data. It is possible for different input data to produce the same hash, however choosing the correct hashing algorithm will mean that chances of that happening are considered too unlikely to be a risk. In this way, it can be compared to a fingerprint. Hashing is also a one-way function - given a hash, it is computationally infeasible (i.e., near to impossible given current computing technology) to calculate the original data, which gives us a secure way to represent a piece of data without using the data directly.

**Public and private keys** also come from cryptography, where the public-private key pairs are used as described in the previous section to scramble and unscramble data.
